# An Inquiry into the Sources and Mode of Action of the Poison of Fever

**Published:** 1841-04-01

**Authors:** Alfred Hudson

**Affiliations:** Physician to the Navan Fever Hospital


### AN INQUIRY

INTO

### THE SOURCES AND MODE OF ACTION

OF THE

### POISON OF FEVER.

By ALFRED HUDSON, M.B. T.C.D.

PHYSICIAN TO THE NAVAN FEVER HOSPITAL.

Much as has been written upon the history of fever, it cannot by any means be considered as an exhausted subject. If indeed we were to test our knowledge of its sources by the universality of their admission, and consider the general agreement of all observers as to their laws as the true proof of these being fully ascertained—a criterion which is applicable to medicine as to the other sciences of observation—we should see reason to conclude that in reality our knowledge of the causes of fever and their modes of action upon the living body is of very small account, and by no means of the most accurate description; for though, in this country at least, the doctrines of localization of fever are not advocated, nor fever considered the effect of inflammation of any particular organ or organs, we find in the most recent writers, equally as in the ancient, the widest differences of opinion as to the phenomena which constitute the origin or nature of this *essential disease.*

An analysis of the mass of conflicting statements upon this subject may perhaps be useful, if only as a preliminary step to other inquiries, by shewing how much of what has been put forward as evidence is really founded on observation, and how much is on the contrary mere matter of opinion and not of fact. Such an examination of what has been advanced upon the disputed question, it is proposed to attempt in the following enquiry.

We regard the essential disease termed fever as the effect of the action on the living body of a morbid poison—in other words of—

"One of that peculiar class of substances which are generated during certain processes of decomposition, and which act upon the animal economy as deadly poisons; not on account of their power of entering into combination with it, or by reason of their containing a poisonous material, but solely by virtue of their particular condition."*

The mode of operation of this poison upon the body is a fertile theme for disputation between the humoralists and solidists of this as of preceding ages, and whence it is derived and where generated—whether in the body or out of the body—the contagionists and non-contagionists are as much disagreed about as ever.

The humoralist holds that the very definition of a morbid poison, if correctly given by the distinguished author from whom we have adopted it, would point to the blood as the subject of its operations. Since, assuming that the *essence* of such poison is that its elements are in a state of decomposition or transposition —and its *action* to communicate that peculiar transposition to the constituents of the body with which it may be brought into contact, *He* finds in the blood a substance the most susceptible of any part of the organism of the action of exterior influences, and whose constituents are the most prone of any to form new combinations. The humoralist also points to the analogy of other morbid

* Liebig's Organic Chemistry.

poisons, which produce their specific effects upon their direct introduction into the blood. He points to the latent period common to both; and, if he be a contagionist (as he must be), he sees in the formation of the poison by the blood the consequence of the introduction of organic matter in a state of progressive transposition or decomposition (such as is the contagious miasm) into a mixed fluid in which its constituents are contained, and the reproduction in that fluid of the exciting body, exactly as yeast is reproduced when added to a mixed fluid in which the gluten from which it originated is contained. On the other hand, the solidist considers that the nervous system is so much engaged in fever, that the poison must be there, or, the phenomena of the latent period are attributable to the nervous system, or, dating this commencement of fever from the nervous shock, sometimes attendant on exposure, and *assuming* that the poison is received into the organism *then* and *there*, he sees an analogy between the action of the poison and certain narcotic substances which he *assumes* act on the nervous system without entering the circulation;—and therefore—fever so acts—or—lastly, the *source* of the poison not being apparent, and the shock preceding the fever, he finds that he can produce contagious fever by a moral impression on the nervous system without the action of a poison at all! These are some of the different opinions maintained by recent and able writers on the nature of fever, and which we shall have to glance at when considering the mode of action of the sources of the disease. As to these sources, our latest writers are so disagreed as to make an analysis of their opinions and evidence no easy matter. If we placed them in juxta position according to the doctrines propounded and denied, our index would run thus—

Fever contagious—not contagious.
Arises from putrefying animal matter—denied.
Arises from putrefying vegetable matter—denied.
Infection a direct emanation from the patient—denied.
Infection capable of being generated de novo—denied.
Atmosphere of patient infectious—denied.
Contact of ditto infectious—denied.
Fomites infectious—denied.
Fever originating in miasm contagious—denied.
Identity of foregoing with typhus asserted and denied.

These conflicting opinions will come under review successively in the course of an examination into the following questions.

1. The existence of a special animal poison arising from infection, and producing a specific disease—typhus.
2. The generation during the decomposition of organic substances of a poison capable of producing fever when applied to the living body.
3. The power of this paludal fever to communicate itself from one individual to another. Does it possess the power of infection per se? in other words, are typhus and typhoid fevers identical? or, does it acquire it by the aid of adventitious circumstances, and so become communicable by conversion into or superaddition of typhus?
4. Arising out of the preceding is the enquiry—what are the adventitious aids to the diffusion of each kind of fever? the laws which regulate their epidemics, and the sanatory measures best calculated to neutralise their operation.

### CHAP. I.—Of the Infectious Animal Poison generated in the living Human Body, and capable of producing Fever when applied to Healthy Bodies.

SECT. 1.—*Proof of its tangible existence.*—It might have been supposed that the accumulated evidence of infection presented in the histories of the typhus of Great Britain, would satisfy the most incredulous; but it is not so, and a recent author has denied the existence of such a cause of fever as atmospheric contagion*—in other words, of " an atmosphere holding in solution a specific contagious poison." Because—" it has never been unequivocally manifested to any of the external senses; it has never been seen combined with the atmosphere, or precipitated from it, or abstracted therefrom to solid bodies."

It has been urged in reply, that this is equally the case with miasm and vitiated air of all kinds, which last, this author himself has endeavoured to prove, is the source of contagious fevers. But this answer is not correct, the fact being, that organic matter in a state of decomposition, or progressive transformation, is present in both. We shall hereafter adduce evidence of this fact with regard to miasm. As to its presence in aerial contagion Liebig states that—" all the observations hitherto made upon gaseous contagious matters prove that they also are substances in a state of decomposition. When vessels filled with ice are placed in air impregnated with gaseous contagious matter, their outer surfaces become covered with water containing a certain quantity of this matter in solution. This water soon becomes turbid, and in common language, putrefies; or, to describe the change more correctly, the state of decomposition of the dissolved contagious matter is completed in the water. The odour of gaseous contagious matters," says the same author, " is generally accompanied by ammonia, which may be considered in many cases as the means through which the contagious matter receives a gaseous form......... Ammonia is very generally produced in cases of disease; it is always emitted in those in which contagion is generated, and is an invariable product of the decomposition of animal matter. The presence of ammonia in the air of chambers, in which diseased patients lie, particularly of those afflicted with a contagious disease, may be readily detected; for the moisture condensed by the ice in the manner just described, produces a white precipitate in a solution of corrosive sublimate, just as a solution of ammonia does..... By evaporating acids in air containing gaseous contagions, the ammonia is neutralized, and we thus prevent farther decomposition and destroy the power of the contagion that is its state of chemical change."

" To this decisive proof of its presence may be added the fact of its being frequently recognized by one of the senses, that of smell, in those cases, in which it has proved active as a poison. For instance—a gentleman in this neighbourhood, not long since, passed through a severe and lengthened typhus fever. About the tenth day of convalescence, while walking across the room, leaning upon the arm of his son, the latter was struck by the odour from his father's body; he immediately became sick at stomach, and on the next day had rigor followed by fever of the same type and duration (21days) as his father's."

Dr. Montgomery's† attack of fever, related by himself, gives similar proof that the aerial contagion may be occasionally recognized by this sense.

" On the 10th of August, I visited a patient in fever, and hearing from the nurse that there were spots on the patient's skin, I stooped very close to her to satisfy myself, and while so doing, I was sensible of a very disagreeable odour from the skin. At the moment, it made a considerable impression on the sense of smell, being almost as pungent as the odour from an ammoniacal salt. The smell continued in my mind all day, &c."

It is true a sceptical reasoner might argue for the possible existence in such cases of an unhealthy locality, impure air, &c.; but much of the evidence of contagion which we possess, is free from any such objection.

---

* Dr. Scott Allison—Essay on Contagion.

† In Marsh's Essay on the Origin of Fever.

Sect. 2.—*The Testimony in proof of the Power of this Poison to cause Fever*—or, as it may be expressed, the proof that the disease has arisen from exposure to the emanations from the bodies of those similarly affected, requires to be of a very exact kind, since the opponents of the doctrine of infection, who, like the writer above quoted, affirm, that "those who have communication with the sick do not suffer in a greater proportion than those who keep apart," explain the many instances in contradiction of this assertion which occur among the medical attendants, nurses, and relatives of the sick, by attributing them to the "locality" and to "impure air," and add, that "it is however almost entirely on such exceptions as these that the contagionists depend for the maintenance of their gloomy doctrines."*

The great weight of the proof derived from the experience of the large fever hospitals in England, Ireland, and Scotland, has been well shewn by Drs. Tweedie,† Alison,‡ Christison,|| and Davidson,§ and the last gentleman justly observes, that "the simple relation of these facts would, with the majority of men, produce conviction that fever was at least contagious in these hospitals, provided the mind was not pre-occupied with an opposite theory." Certainly none but a determined anti-contagionist could resist the fact, that, in the large fever hospitals of the three countries, every clerk has, during some period of his attendance, laboured under fever.

It is also ably proved by Dr. Christison, that the proportion of attacks among attendants is in the ratio of their exposure to the emanations from the sick. It being observed that, in the Edinburgh hospitals, they were affected in the following order as to frequency. 1. Nurses. 2. Resident clerks or house-surgeons. 3. House servants. 4. Medical students not attached to the service of the institution. Thus, in the epidemic of 1818, of 38 nurses, only two or three escaped. Of the 15 gentlemen who filled the office of resident clerk between 1817 and 1820, only two escaped.

But, overwhelming as this argument from hospital experience appears, some have considered it open to objections.¶ It has been argued, that the typhus thus received (or rather the infection of typhus) is *factitious*, and created by causes over which we ought to have exerted due control; "that the poison can only be made effective through contamination of atmosphere under long-continued accumulation of morbific effluvia; and, in fine, that the atmosphere of the patient is infectious, and not his person." This argument receives some support from the experience of large general hospitals, which, particularly in London and Bristol, give admission to cases of typhus without its ever being observed to spread; and, from the acknowledged rarity of communication under the closest approximation among the better classes of society. It is said also that M. Louis never saw a case of communication of fever in an hospital, and Dr. Elliotson states that he never saw a case of fever infectious. It may

---

* Medico-Chirurgical Review, Vol. II. New Series.

† Clinical Illustrations of Fever, and Art. Fever in Cyclop. of Pract. Med.

‡ Essay on the State of the Poor in Scotland.

|| Library of Medicine. Article, Fever.

§ Thackeray, Prize Essay.

¶ Dr. Fergusson, Edinburgh Med. and Surg. Journal, No. 112. See also a Discussion at the Royal Academy of Medicine, reported in the Medico-Chirurgical Review, Jan. 1839, in which the opinion was advocated by MM. Rochoux and Chervin, "that the disease is not communicable directly from one person to another, but is only transmissible in the way of infection, when the atmosphere around becomes loaded with the miasms which exhale from the bodies of the sick."

however be urged in reply, that the observations of the latter eminent observers apply to a different fever—an endemial; and that the argument proves no more than that the infection of typhus is weak, compared with other infections.

But there is another kind of evidence, scarcely less decisive than that derived from the records of the large hospitals. It is thus somewhat flippantly disposed of by Dr. Davidson.

" In the outset it may be stated that we do not mean to fatigue the reader by stories about fomites, and persons who have carried the contagion about them for months or years, nor to hunt out a particular individual who has conveyed it from one town to another," &c.

Now we think it is an admirable rule "as laid down by Dr. Elliotson,"*—" That for infection to be *proved,* the individual who communicates the disease must go from the place where he resides to the spot where the healthy person is, and there give it to the latter. If the healthy person go to the sick person, and the sick person be still in the place where he was living when attacked, then no one can say that the disease which the former contracts has not been produced from the *situation,* and not from the *patient.* The disease may have arisen from contagion—from the emanations of the patient—but this is not proved....... Whenever such a thing occurs as disease being produced in a healthy spot by the approach of an unhealthy person to a healthy one, or by the application of fomites to a healthy person, then it is a proof of contagion, provided the instances be sufficiently numerous, for one or two cases may be quite accidental."

The following is a fair case of importation by both person and fomites :—" A beggar from Limerick obtained admission into a labourer's cabin for herself and a dying child. In five days after she quitted the cabin fever took place in one of the family, which consisted of a man, his wife, and five children, and in succession, within a day or two of each other, every individual sickened, and two children from a neighbouring cabin, who had attended the child's wake, took the same fever within ten days after, and communicated it to their family. The beggar (*herself in good health*) went to a farmer's house two miles distant, and obtained a lodging for the night, after her child was buried—every individual in the family (five in number) also took the fever within a few days—these fevers were all severe."†

That these cases of importation of infection occasionally exercise a very great influence in the spread of fever, we are convinced by our own hospital experience. From the middle of the year 1834 to the same period in the year 1836, scarcely a single case of *contagious* typhus was admitted into the Navan fever hospital. The entire number of *fever* cases only amounting in that time to 363, and these being all instances of epidemic gastric fever or endemial typhoid fever. In the month of July, 1836, three cases of a *new fever* were admitted together. On enquiring their history, I was told that one of them was the seventh of his family who had been attacked—the other six having died. The two men who were admitted with him came from the same neighbourhood—seven miles distant—and had both had communication with the infected family. These were cases of typhus with measly efflorescence, profound adynamia, delirium, &c.

About two months afterwards an elderly man, with six of his family, were admitted labouring under typhus fever. They were from an opposite direction, about seven miles distant. The fevers, of which these were the commencing cases, spread rapidly and widely, and such was their effect upon our admissions, that the number of fever patients increased from 363 in two years to 400 in

* Lectures by Rogers.
† Barker and Cheyne's Report, Vol. II.

1837, and 600 in 1838; when they were at their height. While these cases continued distinctly marked, and differed so much from our ordinary endemial fevers, as to be recognised at once by the nurses, as well as to be dreaded from their greater fatality and until they became merged in the epidemic of the past year, our cases of typhus were nearly exclusively derived from the districts in which these originated, or to which they had spread.

Careful enquiries were made as to the source of the epidemic in each case, and the following particulars were ascertained.

It appeared that in the first, a man had arrived in this country from America. It was stated that the voyage had been an unusually rapid one, and he had been ill the whole, or nearly the whole time. On landing, he was immediately removed to his father's house, twenty miles distant, and on his arrival there, was seen by a medical man, who pronounced his disease to be fever. He died on the second day after his arrival.

His father's house and neighbourhood was previously quite healthy; but in two days after his death, the father sickened, and, on the day following, his sister. She communicated the disease to her husband, who lived half a mile distant. He was attended by his brother, who caught the disease, and was one of the three first brought into hospital. The father was visited before his death by a brother, residing nearly two miles distant—on his return home he sickened, and in the course of his illness communicated it to his son. A brother of the importer contracted the disease, (apparently from his father,) and was sent into hospital, where he died, as did all the above, with the exception of the one who was sent to hospital. In short, of the family of the importer, eight out of nine were infected, and seven died.

In the course of a short time several other families, we have been informed, were completely exterminated. It spread with a rapidity and fatality perfectly unprecedented and long maintained its hold in the town and neighbourhood.

We, of course, have no means of determining the source from which the original case was derived; but we were much struck on meeting with Dr. Gerrhard's account * of the typhus which prevailed in some parts of America in that year, with the resemblance between this epidemic and that which he has so well described—especially in the acrid infectiousness of both.

We have not to *hunt* so far for the second case.

This man's daughter was a servant in Dublin, where she contracted typhus and died. Her brother went to see her, and remained till her funeral took place. He sickened—came home, and died of what was described to me as a long, spotted, fever.

After his death, the abovementioned seven persons sickened within a day or two of each other, and were sent to hospital. The father died; several of the others had very severe fever; typhus spread from this house, first to the immediate neighbourhood, and subsequently to the surrounding country.

These instances are by no means all of the kind that have occurred within the time mentioned, but are selected on account of their wider influence and the unequivocal nature of the testimony they afford to the infectiousness of fever.

Among the most unquestionable sources of fever, is the communication of it by fomites carried from the patient to some place previously healthy.† It is in this manner that washerwomen frequently become the subjects of fever. Dr.

---

* American Journal of Medical Sciences, February, 1837, and Dublin Medical Journal, July, 1837.

† Dr, Stark's Experiments on the Power of different Colours to absorb Odorous Particles, (Edinburgh Philosophical Journal, April, 1834,) shew that woollen substances constitute the most powerful fomites.

Tweedie says, " to shew that the disease may be engendered by fomites in clothing, the laundresses, whose duty it is to wash the patients' clothes, are so invariably and frequently attacked with fever, that few women will undertake this loathsome and frequently disgusting duty."

Dr. Armstrong,* an anti-contagionist, had previously noticed the same fact.

Dr. Reid and Dr. Cheyne,† inform us that, during the epidemic of 1817, not a single person of those appointed to receive the clothes of the sick escaped the disease.

The preventive effects of an early removal of the sick is one of the strongest proofs of infection, since the same measure produces no such effect in the endemial fevers.‡ The effect of early removal of the sick and the cleansing and whitewashing of their apartments, was very remarkable in checking the progress of the disease in some families, while, from the neglect of these precautions, the number of the sick rapidly increased in others. Two neighbouring houses, in Barrack-street, afforded an illustration of this remark, viz. Nos. 41, and 47. In the former the disease began in two different families, and its progress was immediately checked by early removal, cleansing, &c. In the latter, the individual first affected remained at home and died of the fever, but not before he had communicated the disease to eighteen persons in a short time.

On the effect of early removal of the sick, Dr. Alison § remarks, we should have little difficulty in pointing out above a hundred houses where a single case of fever has occurred, where the patient had been removed speedily, and the place cleansed, and where there had been no recurrence, &c. Dr. Ferriar || states, " that formerly, when a fever began in the Manchester Infirmary, it was found necessary to dismiss almost all the patients...... but since a few rooms were built in 1792, separated from the rest of the wards, for the reception of such cases, though the infection has been more than once introduced, yet by removing such patients as shewed symptoms of fever at their first appearance into the secluded ward, and preventing all communication between them or their nurses, and the other patients and servants, the complaint has been stopped; and no reason has again occurred for a precipitate discharge of patients."

But in applying these facts to the proof of the infectious *nature* of fever, we are again met by the argument, that under all these circumstances *of* crowding, &c. they only prove that a factitious atmosphere of contagion is produced, and the anti-contagionist points to instances of typhus received into the Bristol and other hospitals, and mingled among the other patients without ever spreading the disease.

Dr. Davidson's quotation from Prichard, supports this view as regards Bristol. " In St. Peter's the wards are very small, and the beds were near each other —offensive smells, often perceptible, and, under these circumstances, the disease manifestly contagious. In the infirmary the wards are lofty and well ventilated —here also the fever patients were dispersed among invalids of every description—no instance occurred of the propagation of the fever—none of the nurses were attacked, nor any of the patients infected, though lying within two feet of cases of typhus gravior." (page 12.)

From the infectious form of the disease prevailing almost exclusively among

---

* Lectures by Rix.

† Dublin Medical Transactions, Vol. 3; and Dublin Hospital Reports, Vol. 2.

‡ Report of Inspectors of House of Industry, quoted by Cheyne. Dublin Hosp. Rep. Vol. 2.

§ Edinburgh Medical and Surgical Journal, Vol. 28.

|| Medical Histories, Vol. 2.

the poor, it is difficult to obtain, in Ireland, a case not liable to the above objections. The following is perhaps as decisive, and as free from objection as may be.

In the month of March, 1839, an old man, with his son and daughter—all of them persons of cleanly appearance—and in comfortable circumstances—were admitted into the Navan Fever Hospital. The history which they gave of their seizure was, that another son, the only other member of the family, had contracted fever, by sleeping for two nights in a house eight miles distant, in which was a person in an advanced stage of the disease. On his return home, he lay down in a fever of twenty-one days. About the 3d day after his crisis, his father sickened—on the following day, his sister, and in a day or two after, his brother. A day or two before these persons came into hospital, a young man, a cousin of the parties, was admitted. He was one of a family of *ten* living near his uncle's house. He *alone*, of this family, visited his cousin during his illness. His family shewed their caution farther, by sending him into hospital early in the disease. He passed through the same fever (typhus, severe in all, and fatal in the old man,) as the others, but no one of his large family took the disease; and on enquiry, a year afterwards, I learned that they were all still free from fever.

SECT. 3.—*Varieties in the Nature or Sources of the Poison.*—The dogma of Dr. Bancroft, that the contagion of typhus*—" The original work of our common Creator must have been continued in existence by the energies of a living principle, exerted successively in the different bodies through which it has been transmitted from one generation to another,"—has met with comparatively few supporters among late writers on fever. Elliotson,† Barker,‡ Roupell,§ Perry,|| and Davidson,¶ espouse this doctrine, but without adding in the least to the meagre facts upon which it is founded.

On the other hand, numerous observers assert the production of typhus under circumstances in which the existence of a fever poison derived from a person labouring under the disease, was out of the question; and therefore they have assumed " that certain physical and moral conditions may so act on the operations of the body as to cause it to generate within itself that which produces the phenomena of fever, independent of any exterior poison."

Dr. Ferriar ** thus enumerates the circumstances, under the combined action of which fever has been observed to arise spontaneously.

1. Want of fresh air.
2. A deficient or improper diet.
3. Want of cleanliness, and, chiefly, want of a proper renewal and change of clothes.
4. Anxiety and depression of spirits.

The second and fourth of these are probably the essential causes of the generation of the poison, and the others assist by producing its accumulation—as in typhus—the diseased emanations constitute the poison ; which, however, is all but harmless, unless accumulated.

The following graphic sketch of fever, thus originating, is given by Dr. J. Hunter.†† In the month of February, 1779, I met with two examples of fever in the lodgings of some poor people whom I visited, that resembled in their symptoms the distemper which is called the jail or hospital fever.

---

* On Yellow Fever. † Lectures, by Rogers, page 296.
‡ Dublin Medical Transactions, Vol. 2, p. 595. § On Typhus.
|| Dublin Medical Journal, Vol. 10. ¶ Prize Essay.
** Medical Histories, Vol. 1.
†† Remarks on the Jail or Hospital Fever, Medical Transactions, Vol. 3.

It appeared singular that this disease should shew itself after three months of cold weather. Being, therefore, desirous of learning the circumstances upon which this depended, I neglected no opportunity of attending to similar cases. I soon found a sufficient number of them for the purposes of farther information.

It appeared that the fever began in all in the same way, and originated from the same causes.

A poor family, consisting of the husband, wife, and one or more children, were lodged in a small apartment, not exceeding twelve or fourteen feet in length, and as much in breadth. The support of these depended on the daily labour of the husband, who with difficulty could earn enough to purchase food necessary for their subsistence, without being able to provide sufficient clothing or fuel against the inclemencies of the season.

In order, therefore, to defend themselves against the cold of the weather, their small apartment was closely shut up and the air excluded by every possible means. They did not remain long in this situation, before the air became so vitiated as to affect their health, and produce a fever in some one of the miserable family. The fever was not violent at first, but generally crept on gradually, and the sickness of one of the family became an additional reason for still more effectually excluding the fresh air, and was also a means of keeping a greater proportion of the family in the apartment during the day. Soon after the first, a second was seized with the fever, and in a few days the whole family perhaps were attacked, one after another, with the same distemper. The slow approach of the fever, the great loss of strength, the quickness of the pulse, with little hardness or fullness, the tremor of the hands, and the petechiæ or brown spots upon the skin, to which may be added the infectious nature of the distemper, left no doubt of its being the same with what is usually called the jail or hospital fever. It would appear there is no great power of infection in the body alone provided the air be not confined. Remarking on the exemption from this disease which warm countries enjoy, he says,—" On the cold is the cause of the air being confined which gives rise to the poison, and thus, directly opposite to the opinions usually received, there is more danger of producing this disease in a cold country, and in a cold season of the year, than in a warm one."

A person exposed to and living in the poisonous air becomes feeble and irritable, his sleep is disturbed, his tongue is white in the morning, his appetite is impaired, and the smallest bodily exertion quickens his pulse and fatigues him. He will remain in this state for weeks together, without any formed attack of fever; yet another receiving the infection from him, shall suddenly be seized with a violent disease. In this manner it is, I much suspect, that prisoners brought into a crowded court often produced the most dreadful consequences, by disseminating the infection lodged in their clothes. An instance of this kind is given by Dr. Fordyce,* which deserves mention. Arguing for a distinction between this poison and putrefactive poisons, he says—" This is undoubtedly not the case, since infection has arisen from a person brought out of rooms in which numbers had been confined for several months, but kept clean from all putrescent matter, so that there was no particular smell or other sensible quality. In one case that came under the observation of the author, a person under such circumstances, from whom no peculiar smell arose, or any other sensible effluvia, communicated the infection to four others with whom he was carried in a coach for about half a mile, so as to produce fevers in all of them, which fevers were violent and fatal."

Dr. Ferriar properly includes *moral* causes—" because it is not proved that the mere confinement of the effluvia of clean and healthy persons, free from

---

* On Fevers, Dissertation, I. page 114.

mental uneasiness can become poisonous. This view derives considerable support from the following remarkable case by Dr. Harty, of the origin of fever from a single person under such circumstances.

A gentleman* was suspected of having confined and ill-treated his wife. At length two gentlemen, one of them a clergyman, having obtained the necessary authority, visited the house, and examined every apartment for the wretched object of their humane search—at first in vain ; but at length a small closet door attracted their notice, and having insisted on its being opened, both gentlemen eagerly entered, and as precipitately retreated. One was immediately seized with vomiting; the other (the clergyman) felt sick and faint. After a little, they recruited and called the wretched woman from her prison hole, in which she had been for weeks immured. It was a small dark closet without *light* or *air*, and in it she had been immured without a change of clothes. At the end of a week both gentlemen had fever ; both took to their beds almost on the same day. The clergyman died, and the other recovered with great difficulty after a severe struggle. Both cases were alike throughout, except in the termination. The woman had not then or afterwards any febrile disease, and had been free from any at any period of her confinement.

Bursts of fever from this cause occur, at times, in situations where no possibility of contagion from without exists—as in prisons, in surgical hospitals, and in situations in which typhus does not usually prevail and has not been introduced from without. Dr. Harty gives unequivocal testimony of this fact, derived from his experience in the Dublin prisons. For cases occurring in crowded wards of hospitals during cold weather, we may refer to Palloni,† Currie,‡ Tweedie.§

Dr. Ferriar|| gives a decisive instance of fever arising in the habitations of the poor from this cause at Carlisle in 1778—9. We must be content to refer the reader who may be desirous of sifting the evidence on this much disputed question, to the above writers, as a recital of the cases would occupy too much space. It cannot be doubted that this depraved atmosphere has been sometimes considered as a source, when it really only favoured the diffusion of the fever poison, whether emanating from the bodies of typhous patients or from paludal sources. We shall have occasion to recur to this subject when examining the circumstances which favour the diffusion of fever as an epidemic disease. At present it may be remarked that, the writers on both sides of the question, have relied in some instances, upon exceptionable proofs. Thus Dr. Peebles, in his valuable paper, adduces several cases which occurred on board ships, which are seldom free from some of the paludal sources. It is also sagaciously remarked by Lind, that it is in ships going from home, and not in those returning from the longest voyages, that fever is found. The reason is obvious.

But if weak cases have been adduced in proof of the origin of fever from this source, they have equally been relied on by the great opponent of the doctrine and his followers. Dr. Bancroft has rested much of his argument upon the fact, that on board slave ships, where the crowding was unprecedentedly great, fever was unknown.¶

But, as has been well observed by Dr. Fergusson,** there are two good reasons for this.

---

* On Fevers, page 163.

† Quoted by Dr. Peebles, Edin. Med. and Surg. Journ. No. 125.

‡ Medical Reports, page 6. § Clinical Illustrations.

|| Medical Histories, Vol. I.

¶ On Yellow Fever, page 127, &c. It is worthy of notice, that in the passage quoted from Dr. Lind (page 128), the liability of felons in transports to fever is asserted. ** Edinb. Med. and Surg. Journal, No. 112.

1st. The absence of all fomites—the wretches being naked there was nothing to retain the effluvia.

2nd. The high temperature, which is always destructive of the poison of typhus.

The absence of fever from the huts of Fins and Russians, may be explained in a similar way, by the high artificial heat constantly kept up in them, and the total absence of moisture. None of the advocates of exclusive contagion, from Bancroft to Davidson, add any facts to the meagre evidence upon which the argument is founded. The enquiry is altogether one of the most important connected with the subject of fever, and bears strongly in its consequences upon science and humanity.

For if it appear that the poison of typhus can be generated de novo, under the conjoint action of the above-mentioned moral and physical causes, we should institute enquiries as to the part which each performs in the production of this result, and without wishing " *to get rid of a difficulty*," we should, on other grounds than our inability to trace contagion to its primordial source, pursue the investigation of its laws, disregarding any such affectation of strict logic as is contained in the following passage.* It is not intended, however, to enter into any speculations respecting the primordial source of the contagion of typhus, for the sources from which it, as well as that of the other contagious fevers originated, are involved in absolute obscurity; and though we could trace them to the most remote æra in antiquity, the same difficulty would be encountered. Some authors, apparently to get rid of this difficulty, and to account for the occurrence of typhus, where no contagion could be traced, have adopted the opinion, that it may be generated by common causes, such as impure air, filth, &c., and be afterwards capable of propagation by contagion. *The argument of analogy is directly opposed to this belief, for if in nature there be no exception to the law, that two causes are never required to produce precisely the same effect, it will follow that, whatever cause can be best reconciled with the phenomena of typhus, must be considered the true source of the disease.* And accordingly this writer proceeds to return a hasty verdict of " not proven," upon the claims of every cause but this " one true source," *contagion.*

The following remark of the venerable Dr. Stokes upon this subject is too apposite to be passed over without notice. "This supposition of a *single cause* of the effects we witness, is quite unsupported by nature. Every animal, every plant, every rock, requires for its production the co-operation of many causes that we know, and most probably of many more that we have not yet discovered. All nature depends ultimately on a single cause, but it has pleased the Almighty to cause that the effects which concern us immediately should arise from the co-operation of several of his creatures."†

*Again*, if it appears that the febrile poison can be thus generated, we need not follow Dr. Barker‡ to the Continent of Europe to look for it. Nor need we to accompany§ Dr. Lombard upon his geographico-typhoid tour in proof that the frieze coat of the Irish labourer is its depository, in which it is exported like other " native manufactures."

---

* Davidson, page 2. † Essay on Contagion, page 25.

‡ Dublin Medical Transactions, Vol. 2.

§ This notion of Dr. Lombard's, along with an opinion expressed by Mr. Farr, in the article Vital Statistics, in M'Cullagh's Statistics of the British Empire, "that the poor Irish are keeping up, if they are not introducing, the fevers of their wretched country in the heart of the British cities," has been met by Dr. Cowan, and by an acute reviewer in the Dublin Medical Journal, for January, 1838. But the latter, while he confers a merited castigation upon Dr. Lombard, bears too hard upon Mr. Farr, whom he classes with certain humane political economists who wrote, that it would be well that Ireland were sunk in the

But the question has a great bearing upon humanity and political economy. Take the case of an epidemic such as has prevailed in Ireland during the past year. Suppose that in a town containing a great number of poor in which fever perhaps has not yet appeared, the inhabitants meet to confer upon the best preventive measures. These will differ as their views of the sources of the disease differ; one may suppose that the contagion is in all cases *imported,* and can see no protection except in a "cordon sanitaire."

Another believes that fever is exclusively of *endemial origin,* and he says—make sewers, sweep away the dung-hills—white-wash the houses.* While the man *alone* who conceives the generation of the poison under the foregoing circumstances, possible will recommend the true prophylactics, and, by providing clothing and fuel, cause the light and air to be admitted into their crowded dwellings, and by relieving mind and body from the pressure of impending starvation, will both render them less susceptible *of* disease *if* it approach them, and less capable of generating in themselves the poison which he believes may arise among them without exterior communication.

On this question it is impossible to speak of humanity and political economy apart. The following extract from Dr. Alison's essay on the management of the poor in Scotland, will prove how even motives of economy should lead to the application of the true preventive-relief of the wants of the poor.

"'A fever which consigns thousands to the grave,' says Dr. Harty, 'consigns tens of thousands to a worse fate—to hopeless poverty; for fever spares the children and cuts off the parents, leaving the wretched offspring to fill the future ranks of prostitution, mendicancy, and crime.' 'The mortality of fever,' says Dr. Barker, 'is most frequent where it is most injurious, viz. in men advanced in life, the heads and supports of families, the increase of poverty and mendicity, and the agonizing mental distress to which it must give rise, are consequences which must occur to every reflecting mind.' There is no exaggeration in the simple and impressive statement of Dr. Cowan—that 'the prevalence of fever presents obstacles to the promotion of social improvement among the lower

---

sea. And says there is not the slightest evidence that the labouring classes *introduce* fever into the hearts of British cities. Probably not. In the case of Glasgow, Dr. Stuberoh's paper, Dublin Journal, No. 39, would seem to shew that they do not—at least by *importing it.* But in an able and temperate reply in the second edition of M'Cullagh's book, Mr. Farr has shewn that, in the three great avenues by which the Irish labourers enter the kingdom—Bristol, Liverpool, and Glasgow, their crowding to excess in lodging-houses, their loathsome diet and filth, are productive of epidemic fever, and he concludes with the following wise remarks.

"In directing attention to a weighty sanatory fact, it is far from our intention to convey any reflection upon the Irish people. We shall, in treating of epidemics, shew that the English were formerly in as bad a condition as the Irish, and we must say we had imagined that any attempt to prove that England is vitally interested in the prosperity and happiness of Ireland, would be rendering neither country disservice........ Reduce your neighbours to ruin and starvation, and you inevitably give rise to diseases which lower like avenging angels over your own heads....... So God avenges oppression; it reaps the fruits of its own handiwork.—(M'Cullagh's Statistics, Vol. 2nd. p. 529).

* See Sanatory Reports of Poor Law Commissioners, p. 14, and Report of the Select Committee on Health of Towns, p. 111.

Also the following passage from a Report of Dr. Addison's Essay on Malaria, Lond. Med. Gazette, vol. 3, N.S. p. 796.

"He thought that if any *palladium* could be discovered potent for the salvation of the city, it would be found in the shape of a *scavenger!*"

classes, and is productive of an amount of human misery credible only to those who have witnessed it.' In the last situation in which I have seen fever prevailing epidemically in Edinburgh, (new land at the foot of the old fish market close) I find, on enquiry, that five families out of the inhabitants of twelve rooms in the two upper flats of the house, have been rendered fatherless by it." p. 9.

We could parallel these cases in this town, but it is unnecessary. There is one more consideration arising from this subject—it is a selfish one, and therefore not the least powerful—it is contained in the following profound reflection of the excellent Ferriar. "The diseases arising from wretchedness differ in this respect from those of luxury; the first are generally *infectious*, the latter solitary but *hereditary*. This observation would furnish an excellent moral, but as it is needless to suggest it, I pass on to my next point."

### SECT. 4.—*The Mode of Action of the Poison, and the Circumstances which assist its Operation in the Human Body.*

The opinions of the majority of physicians of the present day are divided, as to the *theory of fever*, into two parties—the solidists and the humoralists.

That of the former party is thus announced in the article fever, Library of Medicine, by Dr. Christison. "The theory of fever, then, which seems most consonant with the whole facts, with the general sentiments of the profession, especially in Britain, and with a sound and prudent practice, is probably the following. Fever is an essential or primary disease. The first appreciable event in the chain of sequences constituting fever is a functional injury of the nervous system. The only essential or invariable consequence of this affection is functional derangement of most of the important organs of the body, but more especially of the brain, the circulating organs and fluid, the alimentary canal, and the skin.......... The changes which have hitherto been observed to take place in the blood and other animal fluids, are, like the local disorders, secondary and not primary. They may be the source of the phenomena remarked in the advanced stage of the disease, but they are not the source of the disease itself in the first instance."

If we turn to another recent work of high authority, we find the very reverse order of sequence is maintained. "It appears probable, if not certain, from what has been advanced, that in a certain class of fevers (typhoid) the blood is *primarily* diseased, and that certain changes in one or more organs take place as a consequence or secondary effect."*

It will be seen that neither of these distinguished writers assigns the phenomena of fever exclusively to his system; and it has been well remarked, "that all febrile disturbances are disturbances of such vital actions as are the *joint* product of these two great factors of vital phenomena—for example, the primary phenomena of all fevers are—1, disturbance in the formation of animal heat; 2, disturbance in all the secerning functions; 3, disturbance in the process of nutrition. But the formation of animal heat, secretion, and the nutritive process, are all dependent on the conjoint action of the nerves and blood-vessels. Either of these two systems may receive the first morbific impression, but the one soon participates in the changes of the other."†

This last sentence involves the proper terms of the controverted question, for, while all must admit that the phenomena of fever *established* are due to the conjoint operation of the nervous system and the blood, the solidists maintain that it is upon the nervous system the morbific impression of contagion acts primarily; while the advocate of a modified humoral theory holds that the source and primary seat of typhous fevers, properly so called, is proved to be in

---

* Dr. Tweedie. Art. Fever. Cyclopædia of Practical Medicine.
† Ferguson on Diseases of Women. Part 1. P. 97.

the blood; and that the order of sequence is, first, a vitiation of the blood by the commixture of deleterious substances; next, in consequence of such vitiation, an alteration of the functions of the nervous system; and, lastly, the blood that supports the organs, and the nervous system that animates them, having suffered a general injury, a constant though not always appreciable modification of these organs in their function or in their texture."

The advocates of each theory construe the phenomena of the latent period in accordance with their peculiar views: thus, while the humoralist regards it as the time intervening between the absorption of the poison and the manifestation of its effects on the great nervous centres—the advocates of the opposite theory consider that "the symptoms which characterise this period, whether they be slight, or whether they be severe, indicate a disturbance affecting primarily the nervous system.*

Again,—" We are not of opinion that the time between exposure to contagion and the formation of the disease thereby caused, is a period of health: the nervous system was affected previous to any disorder of the circulating system."†

From these extracts it will be seen that it is to the explication of the phenomena of the access and latent period of fever, and not to the *formed* disease, that each theory is to be applied, and its agreement with these phenomena tested.

This narrowing of the question deprives the humoralist of all support from the fact of changes detected in the blood subsequent to the latent period, since these may be owing to the changes in the nervous system; while, on the other hand, it reduces the available arguments for the nervous theory to two. That from the analogy of the morbific impression of contagion to the action of certain poisons—" such instantaneousness of action being supposed to be incompatible with the previous absorption of a poison into the circulation;" and, that deduced from the fact, that "a single mental shock often produces protracted disease, without the presence of any known source of the febrile poison."

By thus limiting the dispute, much is given up by the humoralist; since he holds, "that the fluidity or diffluence of the blood, and the violent colour observed in typhus, is not the result of the disease, but, on the contrary, that they are the immediate effects of the specific cause of the fever;"‡ while, on the contrary, it is on the phenomena of the access that the very strongest arguments for the nervous theory are founded.

Passing by the many writers who have rested satisfied with stating their opinions of the origin of fever, without giving the grounds upon which they are founded, we shall examine the arguments for the nervous theory contained in Sir H. Marsh's able paper on the Origin of Fever,‖ which are rested upon a number of histories of the access of the disease, which Dr. Tweedie has pronounced to " contain a body of evidence which should alone decide the question of the contagiousness of fever."

It will be our endeavour, as advocating a humoral theory, to shew that the evidence does not support the conclusions of its distinguished author. These conclusions are founded upon a supposed analogy of the morbific impression of contagion (or infection) to the action of certain powerful narcotic poisons which is *supposed* to be exerted upon the nervous system immediately, and not through the circulation. " Though there can be little doubt," says he, "that

---

* Marsh. Dublin Hospital Reports, Vol. IV.

† Barker and Cheyne's Report.

‡ Vide Rostan's Clinical Lectures on Typhoid Fever, in Johnson's Review for January, 1841.

‖ Dublin Hospital Reports. Vol. IV.

prussic acid, when applied to the surface of the body, is ultimately absorbed, yet the rapidity of its action leads to the conclusion, that its first and instantaneous effect is on the nervous system." And Dr. Law, in arguing for a *mental* origin in one of his cases, in which the person was exposed to contagion before and at the time of seizure, says, "How are we to explain the mode of this individual's attack of fever? If we are to suppose it was contracted from exposure to contagion, we would avail ourselves of the argument of the toxicologist, who reasons that, from the very short period of time in which some poisons exhibit themselves in the system, these poisons affect the system through the medium of the nerves, rather than through the circuitous route of the circulation."

This theory of poisons being assumed, the analogy of the action of infection is thus stated by Sir H. Marsh. "From these facts it appears that the poison of contagion produces its effects *with the same rapidity as the narcotic poisons to which we have alluded.* Headache, debility, sickness of stomach or vomiting, are among the symptoms first perceived; these sensations, with the rapidity of an electric shock, are at the instant produced," &c.

This specious argument from analogy will be somewhat weakened by the following considerations:—

1. It is by no means proved, that any poison, however rapid, produces its effects upon the system, without being received into the general circulation, or before it can be carried to the brain through the medium of the circulation.

Müller's* conclusion upon this question is—"These experiments, as well as many others instituted by well-known physiologists, prove that, before narcotic poisons can exert their general effects on the nervous system, they must enter the circulation." And again,—"The rapid effects of prusssic acid can only be explained by its possessing great volatility and power of expansion by which it is enabled to diffuse itself through the blood more rapidly than that fluid circulates; to permeate the animal tissues very quickly, and in a manner independent of its distribution by means of the blood, and thus to produce the peculiar material changes in the central organ of the nervous system more rapidly in proportion as it is applied nearer to it." But even this explanation of Müller's—while it falls very far short of furnishing the desired analogy—would seem incorrect, since Mr. Blake has found that the poisonous effects *of* prussic acid in a large dose introduced into the stomach will not take place so long as the circulation through the vena porta is carefully interrupted. He even found that, on the effects of the poison being produced by removing for an instant the impediment to the circulation, the animal could be recovered upon the circulation being again stopped, though the three drachms of prussic acid was still in the stomach. Mr. Blake's conclusions from his interesting experiments are*—

1. That the time required by a substance to permeate the capillary vessels may be considered as *inappreciable.*

2. That the interval elapsing between the absorption of a substance by the capillaries and its general diffusion through the body may not exceed nine seconds.

3. That an interval always more than nine seconds elapses between the introduction of a poison into the capillaries or veins and the appearance of its first effects.

4. That if a poison be introduced into a part of the vascular system nearer the brain, its effects are produced more rapidly.

5. That the contact of a poison with a large surface of the body is not suffi-

---

* Elements of Physiology, by Baly. Vol. I. p. 246.
† Edinb. Med. and Surg. Journal, Vol. 53.

cient to give rise to general symptoms, as long as its general diffusion through the body is prevented.*

But secondly—*the suddenness of action of the febrile poison is generally speaking only apparent and not real.*

The infection of continued fever (says Christison) is, for the most part, by no means virulent. And again—fever is usually communicated by long exposure to the emanations from the sick, and seldom by any single short exposure, however decided. It is a common notion that single, brief, decided exposures often occasion an attack; and, in support of this notion, reference is made to cases where individuals can trace the infection, as they imagine, to a particular fever patient, by having experienced some very peculiar morbid sensation at the time of exposure. There is much room for fallacy, however, in observations of this kind, and besides their proportion is small compared with the far more numerous instances where no such sensations can be recalled as having ever been experienced.

It is unnecessary, though it would be most easy, to multiply testimony to the same effect. Even Dr. Marsh says very truly, that " by far the greater number of patients labouring under contagious fever, are not at all aware of the circumstances connected with the origin of their complaint; the impression made at the time of their exposure being in general unheeded or forgotten. Indeed the impression is often times so slight, as to lead one to think that contagion does no more than predispose to fever, and determine the nature of the disease, of which, exposure to cold, fatigue, or some such accidental circumstance, is the immediately exciting cause; so that there appears much reason to believe that, many are so mildly affected, that, were it not for the superaddition of an exciting cause, they would altogether escape fever; hence it happens that numbers affected with contagious fever, trace the origin of their complaint exclusively to

---

* Sir H. Marsh states, that in some experiments performed by himself and Dr. Jacob, the poisonous effects of prussic acid were observed to commence in FIVE seconds; there is therefore a discrepancy between his results and those of Mr. Blake, but the following experiment of the last gentleman would seem to shew that even this short time would allow of the entrance of the poison into the circulation.

" A drachm of the strongest liquor ammoniæ, mixed with five drachms of water, was injected into the jugular vein of a dog. A glass rod which had been dipped in hydrochloric acid, was held immediately under the nostrils; four seconds after the introduction of the first drop of the solution of ammonia into the vein, it was plainly detected in the air expired from the lungs, by the white vapours that were formed upon its coming in contact with the vapour of the hydrochloric acid."

Dr. Christison's experiments on prussic acid (at page 657 of his work on Poisons), do not support Sir H. Marsh's views of the extreme rapidity of action of this poison. While at page 660 he admits that every argument but this is in favour of the theory of its action through the blood, *in which* it was detected by analysis in the case of a cat killed in a few seconds by the acid applied to the tongue.

But in the text I have neglected to notice the fact, so prejudicial to Dr. Marsh's analogy, that the blood in these cases of sudden poisoning is *fluid.* We are also told by Dr. Christison, that in cases of sudden death from the emanations from Parisian privies, the blood is found *black* and *fluid.*

A similar effect is observed in cases of sudden death from other kinds of miasm—for an instance from animal putrefaction, see the Medico-Chirurgical Review, for January, 1825; and for an instance from marsh miasm, see Evans on the Endemic Fevers of the West Indies, p. 22.

cold, wet and other exciting causes of the disease, the time and circumstances of exposure to contagion having been entirely forgotten. Cases of this kind, *which are by far the most numerous*, throw but little light on the origin of fever. It is only by a careful observation of facts of occasional and rare occurrence, such as those recorded in this paper, in which the effects of contagion are well marked and striking, that we can hope to obtain certain and satisfactory results."

There is much truth in the foregoing passage, especially in that part of it which assigns to contagion the action of a *predisposing cause;* but how can this view be reconciled with Dr. Marsh's own theory, that the action of contagion is an " injurious impression upon the sentient extremities of the nerves?" and how far is he justified in assigning the *cause* and *commencement* of fever to sudden and brief exposure, even by cases of rare occurrence, (exceptions he admits to the general rule), such as he has collected? These are questions deserving consideration. We shall return to the first when examining the argument for the humoral theory derived from the latency and cumulative property of the poison; but how do Dr. Marsh's cases support his opinions as to sudden exposure being the *cause* of fever? It is obvious that when it is committed, that the general rule is, " that no perceptible impression is made by contagion," we cannot admit the conclusion that the impression was the cause of the disease, except it appears that no other exposure took place; the more so since the medical and other attendants of fever patients in private houses, and where cleanliness and ventilation are properly observed, frequently perceive these impressions—arising from the odour of the patient or his excretions;—such impressions, however *sickening* at the time, seldom leading to any further ill-consequences; but of twenty-two cases adduced by Dr. Marsh, ten were nurses or porters of fever hospitals, seven were physicians, one a clergyman, and one appears merely to have suffered the nervous shock, as fever did not follow.

The remaining three appear to be unexceptionable instances of fever, arising from a single and concentrated dose of the poison, two, if not all of them being cases of communication by fomites, (usually containing a concentrated poison.)

But again, we have to enquire whether the moment of exposure was that of the *commencement* of the fever? since the argument rests mainly on " such instantaneousness of action of the poison as is incompatible with the idea of absorption into the blood." Here we might remark on the rapid diffusion of gaseous poisons through the blood, and appeal to Mr. Blake's experiments in proof that the poison may enter the circulation even before the impression is felt; but admitting that this impression is a purely nervous one—a shock, or " reaction," as it has been termed—" a resistance offered by the vital powers to chemical action"—it is *not* the commencement of fever. For it may end where it began; the impression may not, and very often is not followed by fever; and in many more cases goes off altogether for a longer or shorter period before fever commences. True, it may continue, especially in persons whose imagination has become alarmed—in which case some writers have attributed the imagination to the influence of the poison upon the nervous system—and in a manner hereafter to be explained, it may shorten considerably the latent period; but we repeat, this latent period will be found to exist in any case in which a *previous* imbibition of infection is not to be admitted. " The symptom," says Sir H. Marsh, " which is generally considered to mark the commencement of a febrile movement in the system, is that commotion of the nervous functions which has been technically termed a *rigor*." This commencement of the *febrile movement* is only mentioned in twelve of his cases, and in these it occurred in four at an interval of from one to two days, in six after several hours, and in two only it is said to have come on " a short time after" exposure to the poison.

The third consideration which may be urged against this analogy is, " that the poison with which contagion is compared is not *reproduced*." As this reproduction of contagion is one of the strongest arguments for the humoral theory, we shall not dwell upon it here, but merely observe that the toxicological argument, while it sets up a forced and false analogy with poisons which are not reproduced, strives to weaken and destroy that which naturally exists between the infection of typhus and that class of morbid poisons to which it may be said to belong—the exanthemata. This has not escaped Dr. Marsh's observation, who admits that "the opinion that to maintain a protracted fever, an internal cause of disease (such as absorbed or generated morbid matter) is necessary, would arise from the phenomena which manifest themselves in the course of an exanthematous fever." But he meets this by the second of the objections we have enumerated to the humoral theory.

"Yet that to excite and maintain continued fever, an *abiding* cause is *not* necessary, might be proved in various ways, but the fact that a *single* mental shock often produces protracted disease, is decisive upon this point."

As Sir H. Marsh adduces no fact in support of the above assertion, turn we to another able physician who, in a recent paper adduces seven cases from his own experience, in proof of the opinions expressed in the following passages : *

"We quite agree in the wisdom of the precaution of satisfying the absorbents, but deny that they are more the channels through which the morbific matter enters the system, in this instance, (fever from contagion), than they are in other cases where there is no reason to suppose either that they are in an unusual state of activity, nor if they were, can we discover any contagion to serve as a *materies morbi* for them to exercise themselves upon. These are cases in which a strong moral impression acts as a direct and immediate cause in the production of a fever, similar in all respects to one from contagion," &c.

And again :—

"We shall proceed to detail some cases of fever which seem to us calculated to throw some light upon the mode in which the first morbid impression is made upon the system in the production of the disease; and see how far these cases tend to confirm the opinion that fever is the result of a miasma conveyed to the system by the absorbents: *or* if it be not, in some cases at least, the effect of a *moral impression* acting upon the nervous system, and exhibiting itself in symptoms indicating a derangement of the functions of this system."

The advocate for the theory of absorption may reasonably require that in such cases the *materies morbi* shall not appear to have been within reach. But of five cases the subjects were exposed to infection, at or before the seizure. The sixth was not (as Dr. Law admits) a case of fever; and we have only one in which fever followed a mental shock, without evidence of infection at the same time existing. To explain away this case, a determined opponent of the nervous theory might adduce evidence of the general diffusion of the fever-poison through the atmosphere of a city, when fever is prevalent in it; he might maintain that at such times †"certain changes take place in the constitution of the atmosphere imperceptible to our senses, and eluding chemical tests, which predispose human bodies to febrile diseases in such a way, that circumstances which in ordinary times would only give rise to a catarrh, an attack of rheumatism, or even occasion no indisposition at all, will now in many individuals become the exciting causes of continued fever."

If it be said that this is begging the question, the humeralist takes higher ground, and asserts that such cases, instead of disproving, strengthen his own theory; inasmuch as he can shew that fever follows strong nervous impressions, in consequence of their lowering the vitality of the blood, and so favouring the transformations in that fluid upon which fever depends. He believes that ‡"no other component part of the organism can be compared to the blood in respect of

---

* Observations on Fever, by Dr. Law. Dublin Med. Jour. Vol. XIV.

† Prichard, on the Epidemic Fever of Bristol. ‡ Liebig, p. 360.

the feeble resistance which it offers to exterior influences. The blood is not an organ which is formed, but an organ in the act of formation; indeed, it is the sum of all the organs which are being formed. The chemical force and the vital principle hold each other in such perfect equilibrium, that every disturbance, however trifling, or from whatever cause it may proceed, effects a change in the blood. Every chemical action propagates itself through the mass of the blood; for example, the active chemical condition of the constituents of a body undergoing decomposition, fermentation, putrefaction or decay, disturbs the equilibrium between the chemical force and the vital principle in the circulating fluid: the former obtains the preponderance. Numerous modifications in the composition and condition of the compounds produced from the elements of the blood, result from the conflict of the vital force with the chemical affinity in their incessant endeavour to overcome one another."

He admits that * "perhaps there are cases in which the modification of the blood is only secondary to a modification of the nervous system. If, for instance, under the influence of a strong mental emotion, this system being suddenly perverted in its action, ceases to exert its proper influence over the different organs in which the blood is elaborated, deposited, and receives new materials, must not that fluid itself become altered in its turn? If so, thence must arise a number of organic and functional derangements varying greatly, according to the mode and intensity of the primitive alteration of the innervation. In such cases we may observe to occur sporadically those same diseases, typhoid or other, that we have just now seen prevailing epidemically under the influence of manifest causes of infection of the blood."

To prove that Dr. Law's case belongs to this formula, let us place it by the side of another in which *precisely* the same mental impression, acting more intensely, produced death. Eliza J——, æt. twenty-six, was admitted under Dr. Law's care, March 28, 1836. She had been in perfect health a week since, when, on missing a piece of linen which had been committed to her care to make shirts, from the apprehension that her honesty would be called in question, she was seized with a violent rigor and sickness, which confined her to bed ever since. Petechial fever, with prominent hysterical symptoms, followed. She recovered with difficulty and slowly.

Sometime ago, I was present at the examination (*post mortem*) of a man who died suddenly under the following circumstances.

He had committed a very trifling theft, for which he was apprehended and carried before a magistrate. He was a person rather above the lower order, and manifested great shame and grief at this exposure. While sitting before a table waiting for his case to be called on, he leaned his head forward on the table and was observed to snore; in a few minutes, the sound of his breathing ceased, and on raising his head, those near him found that he was dead. It was supposed, that apoplexy was the cause of death, and the brain was first examined. It was, however, perfectly healthy. The other viscera were then carefully examined. The only one which discovered anything which could account for his sudden death, was the heart, which was distended with *dark fluid blood.*

Let us suppose that the mental impression had not been so intense in this case, and the life of the blood not so completely and suddenly destroyed,—what would have been the probable consequence? This question is answered by a comparison of the two histories. In the last, the vitality of the whole circulating mass was destroyed, and the symptoms were those of a brain suffering the influence of *a strong narcotic poison.* In the other, the livid, petechiæ, spongy, and bleeding gums, &c. showed to what an extent the vitality of the blood had been destroyed, The immediate occurrence of a rigor shewed that the self-generated poison had

* Andral, Pathological Anatomy, Vol. I. p. 671.

reached the nervous centres, and that the struggle had commenced which was to end with either the death of the whole mass of blood or the elimination from it of the portion so affected. It is worthy of remark, (and is noticed by Dr. Law) that the rigor was immediate,—not after an interval of hours or days, as in cases of exposure to infection, in which the operation of the poison is gradual and often (generally, indeed,) accumulative.

In fine, typhus, or a disease resembling it, but differing, according to Dr. Cheyne, in the very important particular that it is not communicated by contagion—in other words, that the poison is not *reproduced*—is but one of three modes, or degrees, in which the blood suffers from a strong mental impression. It may be killed at once, or it may suffer in a degree insufficient to produce *formed* disease—loss of appetite and depraved secretions, with slight derangements of animal heat, being perhaps the only indications of the injury it has received,—or it may act upon the system in a manner similar to the fever poison. But this cannot be said to prove that the fever poison acts by producing a moral impression; and, therefore, instead of agreeing with Dr. Law, that "even in cases where there was most reason to suspect absorption, where a person having exposed himself to contagion, fasting,—and then contracted the disease,—even here the symptoms exhibited by the disease so resemble those where there is no possibility of suspecting infection, that we cannot but believe that the mode of absorption is the same in both cases, and that as it is not absorption in the one case, neither is it in the other,"—instead of going to this length of denying the existence of a *materies morbi* altogether, we would reduce the two cases to the same formula by an opposite method. As thus: violent nervous shocks kill the blood or modify it, and occasionally produce fever. Contagious and other miasms also, in some rare instances, kill the blood, and, in general, modify it, so as to produce fever. But they may do so without causing a nervous shock. Therefore, they act *directly* on the blood, by being absorbed into that fluid and not through the intervention of any derangement, functional or otherwise, of the nervous system.

The principal arguments for the nervous theory derived from the mode of access of fever, having been examined, we shall submit some of those which tend to support a modified humoral theory, and then offer a rationale of the action of the *causes* of fever in accordance with this theory.

The explication of the accession of the disease having been taken as a text of the opposite thories, we are deprived of any support from two arguments which have been much used by humoralists: viz. the changes which the blood undergoes in the course of fever, and the production of fever or a disease perfectly analogous, by the introduction of substances into the circulation.

Another argument, of a similar kind, is derived from the known power of sources which ordinarily produce fever, to kill the blood at once when their poison is introduced into it in sufficient quantity. We give the fact on the highest authority.* The inference has been met by the toxicological argument already considered, and by a distinction asserted between mephitic poison and the fever poison. This distinction we shall examine along with the source itself, hereafter.

But there are certain peculiarities in the action of the febrile poison which in their general character resemble other morbid poisons, and favour the idea of its absorption into the blood.

The first of these is its occasional latency in the system, in which it will lurk for a longer or shorter period, until called into action by some accidental cause.

"In several instances," says Dr. Graves,† "I have observed that certain diseases, which seemed to have been lurking in the constitution, may suddenly

* Christison on Poisons, p. 700, 2nd edition.

† Lectures, London Medical Gazette, vol. III. N.S. p. 186.

make their appearance in consequence of the operation of causes apparently unconnected with the disease in question ...... I have witnessed several bad cases of bad secondary venereal, in which the attack was traced to excessive fatigue, or a common cold. You will also meet numerous examples of an analogous fact among fever patients: examine them, and you will learn that in a majority of cases their disease arose from exposure to cold. One person fatigues himself by too much exertion in business, and gets an attack of spotted fever; another attributes his disease to over-anxiety; some to intemperance, and some to fright. In all these cases, it is very probable that the poison of fever has been lurking for some time in the system, and has been called into active existence by the operation of some sudden accidental cause, as fright, fatigue, intemperance, or cold."

Something similar, Dr. Graves justly observes, is remarked in the case of the Irish labourers employed during summer and autumn among the fens of Lincolnshire (and we may add Cambridgeshire). During their stay in England, they appear free from disease; but on their return home, if they happen to be exposed to wet, fatigue, or the derangements of health consequent on intemperance, they are very often seized with intermittent fever.

He continues, "Does it not often happen, that many of us escape fever although exposed to its contagion month after month? Do we not go on for years untouched, although subject every-day to the imbibition of the poison? and do we not, rendered bold by our impunity, consider ourselves, as it were, fever proof, until some accidental cause convinces us of the contrary, by giving rise to a sudden and violent attack? Who is there that has not observed this repeatedly among the students attending a fever hospital?"

Similar proof of the latency of the fever poison is afforded by the cases recorded by Lind, of sailors, who apparently escaping from the fever which was raging on board, went ashore, and in some time afterwards, in consequence, apparently, of exposure to cold or debauchery, were attacked, *not* with the fever prevailing there, but with that of the ship they had left. In this respect, then, the febrile poison resembles other morbid poisons.

Again: *it is a cumulative poison.* The exposure of a single moment is probably insufficient, in any case, to cause fever. A few inspirations may accumulate sufficient in cases of great concentration of poison; but there is abundant proof that daily and continued imbibition of the poison is, in general, requisite. Thus, we find the attendants on the sick attacked in proportion to the frequency of their approaches to the infection, the very reverse of what would be the fact if the poison were *not* cumulative, since it is a law constantly observed, that agents which act by single impressions lose their power of producing those impressions in proportion as they are frequently repeated. It is true that some eminent writers aver this of infection, as Dr. Copland, who says, "when a person has escaped infection upon the first or the earlier exposures to several infectious maladies, he will generally continue to possess an immunity, unless circumstances should occur to increase his predisposition." Observations made on a large scale, however, tend to disprove this, as regards typhus.

Thus, when fever prevailed during the retreat of the British army through Holland, we are informed by Dr. Fergusson,* that few, indeed, of the medical staff escaped the typhoid contagion; and, again, in the retreat from Talavera to the confines of Portugal, it was seen that the *best seasoned* of the medical staff were the principal sufferers. Dr. Christison, too,† (a solidist) maintains that it is not improbable that the severity of the disease bears some proportion to the *amount* of exposure."

And ......" In many instances, fever breaks forth apparently from gradual

---

* Edinb. Med. and Surg. Journal, No. 112.

† Library of Medicine, Art. Fever.

charging of the constitution under *constant exposure* to the morbid emanations and without any other co-operating cause."

This is very like humoralism, as is the illustration given by Dr. Haygarth. "A pint of yeast will excite fermentation in a barrel of ale, but a hundredth or a thousandth part would not have the same effect."

Again. The *reproduction of the poison of contagion*, is a fact "not dreamt of" in the philosophy of the solidists. Here their analogy is at fault, for the poisons from whose action it is derived are not reproduced. Neither will any supposable impression upon the nervous system explain the continued reproduction of the same febrile phenomena, and the same miasm through an indefinite series of individuals. We have admitted the production of fever by a strong mental impression. We have endeavoured to reconcile this occurrence with the theory which refers the source of fever in all cases to the blood. We have, however, noticed the fact, that such fever does not reproduce itself, and referred to the testimony of one, whose accuracy of observation has seldom been surpassed, who says, "The most remarkable part of the disease is that it does not spread. I have no recollection of a second case of this kind of fever occurring in a family."*

But the humoral theory has its analogy for the reproduction of the poison.† "The mode of action of a morbid virus, exhibits such a strong similarity to the action of yeast upon liquids containing sugar and gluten, that the two processes have been long since compared to one another, although merely for the purpose of illustration. But when the phenomena attending the action of each respectively, are considered more closely, it will in reality be seen that their influence depends on the same cause."

Now, when yeast is introduced into a mixed liquid, containing both sugar and gluten, such as wort, the act of decomposition of the sugar effects a change in the form and nature of the gluten, which is in consequence also subjected to transformation. As long as some of the fermenting sugar remains, gluten continues to be separated as yeast, and this new matter, in its turn, excites fermentation in a fresh solution of sugar or wort. If the sugar, however, should be first decomposed, the gluten which remains in solution, is not converted into yeast. We see, therefore, that the *reproduction of the exciting body* here depends:—

1. Upon the presence of that substance from which it was originally formed.
2. Upon the presence of a compound, which is capable of being decomposed by contact with the exciting body.

If we express, in the same terms, the reproduction of contagious matter in contagious diseases, since it is quite certain that they must have their origin in the blood, we must admit that the blood of a healthy individual, contains substances, by the decomposition of which, the exciting body or contagion can be reproduced. It must further be admitted, when contagion results, that the blood contains a second constituent, capable of being decomposed by the exciting body. It is only in consequence of the conversion of the second constituent, that the original exciting body can be reproduced.

When a quantity, however small, of contagious matter, that is of the exciting body, is introduced into the blood of a healthy individual, it will be again generated in the blood just as yeast is reproduced from wort. Its condition of transformation will be communicated to a constituent of the blood; and in consequence of the transformation suffered by this substance, a body identical with, or similar to the exciting or contagious matter, will be produced from another constituent substance of the blood. The quantity of the exciting body newly produced, must constantly augment, if its further transformation or decomposition proceeds more

* Dr. Cheyne's Account of Fever from Mental Causes, in Sir H. Marsh's Paper on the Origin of Fever.

† Liebig.

slowly than that of the compound in the blood, the decomposition of which it effects."

These substances are the organic matters existing in the blood, either in the state of transition from blood into the constituents of the tissues, or from food into blood. Which changes, it is argued, cannot take place without the formation in the blood of new compounds, which require to be removed by the organs of excretion.

"When the organs of secretion are in proper action, these substances will be removed from the system; but when the functions of these organs are impeded, they will remain in the blood, or become accumulated in different parts of the body. The skin, lungs, and other organs, assume the functions of the diseased secreting organs, and the accumulated substances are eliminated by them. *If when thus exhaled, they happen to be in the state of progressive transformation, these substances are contagious, that is, they are able to produce the same state of disease in another healthy organism, provided the latter organism is susceptible of their action; or in other words, contains a matter capable of suffering the same process of decomposition.*

"In the abstract chemical sense, reproduction of a contagion depends upon the presence of two substances, one of which becomes completely decomposed, but communicates its own state of transformation to the second. The second substance thus thrown into a state of transformation, is the newly-formed contagion.

"The second substance must have been originally a constituent of the blood; the first may be a body accidentally present.

"If *both* be constituents indispensable for the support of the vital functions of certain principal organs, death is the consequence of their transformation. But if the absence of the *one* substance, which was a constituent of the blood, do not cause an immediate cessation of the functions of the most important organs, if they continue in their action, although in an abnormal condition, convalescence ensues. In this case, the products of the transformations still existing in the blood, are used for assimilation, and at this period, secretions of a peculiar nature are produced."

Having submitted this chemical analogy of the reproduction of contagion in the words of the highest living authority on animal chemistry, it only remains to attempt a rationale of the action of the causes of fever, in accordance with its principles, which may be thus stated:—1st, That the principal character of the blood consists in its component parts being subject to every attraction; the chemical forces of this fluid, and the vital principle holding each other in such perfect equilibrium, that every disturbance, however trifling, or from whatever cause it may proceed, effects a change in the blood.

2nd. That bodies, the elements of which are in a state of decomposition or transposition, when produced from the blood, as contagions are, will communicate *their state* to the sound blood, exactly as gluten in a state of decay or putrefaction, (yeast) causes a similar transformation in a solution of sugar and gluten (wort.)

Assuming then, that the primary action of the febrile poison is upon the blood, there can be but one *essential cause* of fever, viz., *The introduction of the poison into that fluid.* Its activity, or the occurrence of the peculiar transformations which it has a tendency to excite in the blood, will be determined by the existence of certain accessory or accidental causes, which disturb the equilibrium between the chemical forces in the blood and the vital influence; either by their action on the blood, causing the increase of compounds subject to those transformations which the poison produces—as depraved diet, bad air, &c.; or by their action on the nervous system, withdrawing permanently or temporarily more or less of its influence, and so favouring the chemical action of the poison. Such are the depressing effects of cold, fatigue, anxiety, debauchery, disgust, fear, &c. These are usually termed *exciting causes*, the former *predisposing causes*.

The occurrence of fever—the length of the interval which may elapse between the imbibition of the poison, and the first febrile movement; in other words, the length of the latent period—the severity of the disease, and the facility with which infection is received and communicated, will depend upon the relative power of the poison, and its combination with one or more of the foregoing predisposing and exciting causes.

Thus, the continued imbibition of the poison will sometimes, apparently without the co-operation of any accessory cause, result in an attack of fever. This, however, is a very rare case, as though deranged health, and particularly disorder of the receiving functions, may exist, the poison is in this case usually eliminated from the blood, unless the balance of forces in that fluid be disturbed by some one or other of the exciting causes.

The occurrence of the exciting cause may be, or may not be, accompanied by exposure to contagion. In the case of nurses, and the other attendants of the sick, some single exposure being marked by the presence of an exciting cause, it has been supposed that the infection was then and there received into the system, when, in reality, it was before latent, and only rendered active by the *circumstances accompanying* this particular exposure. Again, when the occurrence of the exciting cause is not attended with exposure to infection, the fever is often wrongly attributed to cold, excess at table, mental emotion, &c., the latent presence of the predisposing contagion not being recognised by the patient, and sometimes, as we have seen, being denied by the physician.

The exciting cause may act, not only by determining the occurrence of fever, but also by shortening its latent period.

This is a frequent effect of exposure to infection. "In these cases, the ascertained laws of incubation," says Fergusson, "will so far be set at nought, that a terrified patient will not only fix the precise moment of infection, but will actually sicken prematurely with small-pox, (a latent infection must of course have been previously received), through the spectacle of the disease in the person of another, or through the disgust (and nothing worse), of an excremental smell, strongly affecting his alarmed imagination, or through the same impression, he may fall down the victim of an impossible contagion, like that of yellow fever."

The apparent shortening of the latent period of morbid poisons, seems to occur under these circumstances:—

1. A strong impression made on the nervous system at the time of exposure. If this be so powerful as to affect seriously the vital principle, the effects of the poison will follow with proportionate rapidity. The poison of ague, usually so long latent, affords a good illustration:—Dr. G. Bird relates, "that being employed in some experiments upon the gas in marshes (near Woolwich), having suddenly disengaged a quantity of most offensive gas, he was seized with nausea; and on the day following, with intermittent fever."

A similar instance, in his own person, is related by Mr. Evans; and another, in which death followed in forty-eight hours. *In this last case, the blood was found fluid.**

2. A less powerful impression upon the nervous system may accompany exposure, and be followed by a latent period, apparently shortened, but admitting of the supposition of infection previously latent. Several of Sir H. Marsh's cases afford illustrations of this fact. And it is very probable that exposure to contagion in this way, often produces merely the same effect as an exciting cause, that cold, or any depressing agent would exert.

3. The circumstances accompanying exposure to one kind of poison, instead of acting as accessories to the action of that poison, may cause the immediate action of another, previously latent.

This is the only reasonable mode of explaining the cases of irregular contagion,

* On the Endemical Fevers of the West Indies.

related by Marsh and others, of typhus, received from small-pox patients, scarlatina from typhus, *ague* from typhus, and typhus from puerperal fever.

Some of these cases we might truly term *impossible* contagion, unless explained by the supposition of a previously latent poison. The facility of reception of the disease depends upon two conditions; 1st, the presence in the blood of compounds capable of undergoing the transformation of the poison. This constitutes susceptibility; and when it exists in a great degree, and conjoined with, 2nd. diminution of the vital influence, it constitutes the highest degree of predisposition to disease. The proneness which the living body may thus acquire to infection, may be so great (as seen in crowded collections of wretched beings in large cities, deprived of air, light, fuel, clothing, and sustenance), as to resemble that incapacity of resisting the progress of decay, (a true contagion) which is exhibited by dead animal matter, placed in a putrefying atmosphere.*

The severity of the disease depends partly on the above circumstances, but principally on the *dose* of the poison. This may be illustrated by comparing small-pox and measles, received in the natural mode, with the same diseases communicated by inoculation. Individuals may suffer as severely from the latter as the former, but the generality of persons do not. The following passage in a recent work of great ability offers inducements to consider this subject somewhat in detail:—" The modifications in disease dependent *on the mode of introduction* of the morbific cause, is, however, a subject too difficult for me to grapple with, and the observations are too few too offer any precise result. Cruveilhier, in the article ' Phelibitis Dict. de Med. et Chir. Prac.' points out the increased intensity of effect when pus is introduced into the circulation at once, and as compared with that caused by gradual absorption from an abscess. The modification which small-pox undergoes by inoculation, as compared with that malady acquired by inhalation, is very remarkable."† From this last observation it would appear that the author considers the modification of small-pox as not *consistent* with Cruveilhier's observation. Such an idea must have arisen from confounding the *matter* of the small-pox pustule with the *poison* of small-pox,‡ when in reality it only contains the poison in common with the blood and all its excretions.

---

* A fact noticed by Parent Duchatelet, in some infectious places in Paris; and by Senac, see Wilson Philip on Fevers, Vol. I, page 210.

† Ferguson on Diseases of Women, page 104.

‡ The distinction between them is well stated by a writer in the ' Edinburgh Medical and Surgical Journal,' Vol. LIII, p. 206.

" Rayer mentions ' pus and miasm' as two distinct agents which should never be confounded. If the contagious effluvium and the matter of the pustule were one and the same thing, how could we account for the circumstance of the *fœtus in utero* becoming affected with the small-pox? Besides, Dr. Waterhouse and others have recorded cases in which persons exposed only to the exhalations from the blood of small-pox patients have been afterwards attacked by the disease."

The fact marked in italics would also serve to prove the distinctness of the poison from the ponderable matter of lues. Other considerations would lead us to extend it to all morbid poisons. For

1. The peculiar action of a morbid poison on the blood presumes its possessing great diffusibility in that fluid; and this quality is known to exist in all substances universally, as the cohesion of their atoms, or in other words, their ponderability.

2. The power of permeating tissue depends upon the same condition; and while we find that all the morbid poisons *may* act without abrasion of surface, we find that those which do appear to permeate the skin, act with more certainty if presented at a temperature which admits of their volatilization. This is noto-

The poison of small-pox is equally subtle and imponderable with the other morbid poisons, *an aura*, present it is true in the matter of the pustule, but equally present and equally capable of communicating the disease in the gaseous exhalation which arises from the blood drawn from a variolous patient. The same is

---

riously true of small-pox, as the dissection of subjects who have died of this disease, though not harmless, is much less infectious than the handling of the living body. It is also well known that the examination of any dead body is most likely to be followed by the bad consequences of a dissecting wound, when the body is warm and contains the halitus of its cavities uncondensed......... the next in point of danger being that which is next in diffusibility; the exposure of the surface of the hands to the liquid contents of the serous cavities in particular cases, especially in puerperal peritonitis.

I need only refer to Mr. Stafford's paper on this subject in the 20th Vol. of the "Medico-Chirurgical Transactions" for instances of the imbibition of this poison in puerperal and other cases, without any abrasion of surface. The following circumstance bearing on this subject occurred to myself a short time since.

I was sent for to see a lady in the latter end of her first pregnancy, who I was informed had been for some time suffering much painful anxiety of mind and fatigue of body, and had been laboriously occupied with the arrangements for entering on a new residence, which had kept her constantly upon her feet. For some weeks the legs had swelled considerably, and pitted under pressure. This swelling had rather suddenly increased, and extended to the thighs and pelvic region, with a feeling of stiffness and inability to walk up stairs. Her pulse was quiet, tongue clean, and general health apparently perfect. This was on the morning of the 21st of December. All appeared to go on well, and the swelling seemed to diminish a little till the night of the 23rd, when she slept none, and was attacked with vomiting. On the morning of the 24th I found her remarkably changed; the countenance haggard and anxious, with a quick irritable pulse, thickly furred tongue, restlessness, and vomiting of a dark green fluid. Labour pains came on at 10 a.m. and continued regularly during the day. About 10 p. m. she had an attack of convulsions, and in a few minutes another. Delivery was immediately effected by the assistance of the forceps. It was observed that the labia had since morning become very dark coloured, and the perineum tore upon the slightest stretching like wet brown paper, but *without bleeding*. The delivery of the child was followed by that of a second, unassisted; both being quite dead and flaccid. The uterus contracted firmly, and there was no hæmorrhage; but the patient became less and less capable of being roused, the abdomen enormously distended, respiration laborious, and she sunk at 2 a.m. three hours after delivery.

About four in the evening of that day, I felt a hot painful itching upon the back of my right hand, where I perceived a small transparent vesicle. In a couple of hours I had pain in the axilla, and an uncomfortable, chilly feel. I applied a number of leeches to the hand and took an emetic, followed by calomel and James's powder. These means removed all unpleasant general symptoms, but the part itself did not recover so speedily, as an ill-conditioned obstinate sore formed on the hand which was long in healing. Not the slightest scratch or puncture existed before the application of the poison.

But whence this poison? It was ingeniously suggested to me by my friend, Dr. Clifford, who assisted me through this most distressing case, that the vital powers being over-taxed for the nutriment of two children, had given way, and this decomposition before death was the consequence. Perhaps this is the only explanation which can be admitted.

But I am inclined to believe that the whole was the effect of phlebitis, by which a morbid poison was generated, which produced *death in the fœtuses*, distrue of measles, which has been propagated over and over again by Home, and others, by inoculation with the *blood* of the patient, and with the same result,—a milder form of disease. By thus separating the poison from its vehicle, the difficulty of explaining the modification of these diseases by inoculation is got rid of, since, to recur to the simile of Dr. Haygarth, a hundredth part of a pint of yeast will not excite fermentation in a barrel of ale, though a pint will do it; and it must be obvious that a single inspiration in the immediate neighbourhood of a small-pox patient may introduce more of the *aura* into the blood than the direct introduction by inoculation of an atom of matter, in which only a small proportion of the poison can be present. The correctness of this view could be readily tested; and if it were found that, as in typhus, the amount of exposure had an influence in determining the severity of the attack of small-pox, the explanation must be admitted. One fact is strongly presumptive in its favour; it is the less complete removal of the susceptibility to the disease after inoculation than after natural small-pox. The analogy to the fermenting process is too obvious to need suggestion, and the same remark holds good of fever, short and mild fevers being notoriously more prone to recurrence than a long and severe form of the disease.

---

organization in the mother, and being presented under circumstances favourable to absorption, rapidly permeated the skin to which it was (only for a few moments) applied. Every thing was favourable to the occurrence of crural phlebitis and to the absorption of the poison into the patient's system, as will appear from the history of the case, without again enumerating particulars. Dr. Wilson's paper, in the "London Medical Gazette" for April 1838, proves that crural phlebitis in women is not confined to the puerperal state.

*Note.*—It was not till after the section upon the Theory of Fever had been sent to press, that I met with Dr. Hodgkin's remarks upon the nature of the fever in his recently published volume on diseases of the mucous membranes Lecture 23rd. "I shall now proceed to state what I have conceived to be the condition of the system which constitutes fever, whether it be produced by the influence of some local inflammation or lesion, or exist by itself, independently of such exciting cause. This latter form, however, if it have an existence, I regard as of much rarer occurrence than has generally been supposed. *Fever, I imagine, to depend on the suspension, or at least very considerable interruption of that process by which during health, the various parts of the system are continually undergoing a change,* the old materials being removed, whilst others are substituted in their place. . . . . The process of incessant and universal change of the particles constituting our frames is what we imply by the terms nutrition and "interstitial absorption," it is not merely in its character closely allied to secretion, they are, I believe, essentially parts of the same function," &c. This view is supported by strong facts derived from the phenomena of fever, and by much ingenuity of reasoning. And Dr. Hodgkin proves, *at least*, that such an arrest of the molecular change takes place with reference to the secretions and nutrition of the body in fever. Thus far, there is a coincidence between his theory and that advocated in the foregoing section; the same suspension of secretion and accumulation of organic matters in the system, being part of both explanations, and the phenomena of solution or crisis being explained similarly in both.

The difference is as to the *initiatory movement.* While Dr. Hodgkin would consider the *factor* of the disease to be in all cases a local lesion or inflammation, the theory of a morbid poison supposes it to be a *molecular change in the blood* caused by the dynamic force of the decomposing particles of the poison, from which arise disturbance of the process of innervation, and of the molecular changes of nutrition, interstitial absorption, and secretion.

The theory does not assume to *determine* whether the changes in innervation (such as rigor) which mark the commencement of *formed* fever, are the direct effects

SECT. 5.—*The Characters of the Disease produced by the Infectious Animal poison of Typhus.*

The argument for the foregoing theory of fever, would obviously be much strengthened if it could be made to appear that the phenomena of Typhus are so analogous to those of the other morbid poisons, as to entitle it to a place among "those special contagions, which do not amount to more than five or six, and are all comprehended under that class of which it is the general distinguishing characteristic to occur once only during the life-time of the individual;" in other words, to be classed with the exanthemata.

We find medical writers much divided upon the question whether the petechial eruption of typhus is a primary and essential, or a secondary and accidental character of the disease. We may refer to De Haen,* Hoffman, and especially to Bruserin's elaborate argument for the former opinion, and for the consequent classification of typhus among the exanthemata; and among more recent writers the same view is ably supported by Dr. Copeland and Dr. Peebles, Dr. Roupell, and Dr. Davidson. Dr. Alison seems inclined to adopt it, though his language is reserved and cautious. "Such cases of spotted fever may be said to form the link that connects the order of fevers with that of the contagious exanthemata."†

If it be found that the analogy is complete in every essential particular, and that the objections which have been urged against the classification of typhus with the exanthemata are founded upon supposed discrepancies, which have no real existence, we shall be entitled to substitute for this cautious approximation, the decided definition of Dr. Peebles: "This contagious febrile eruption is an exanthematous affection, the production of human effluvia where society is placed in circumstances favourable to its developement, and should be considered the effect of a poison *sui generis*. It arises from a miasm, which generates in the human body *an eruptive fever distinct from all others, as other exanthemeta are distinct*."‡

The first point of resemblance, and one much insisted on by the older writers, is the *primary* nature of the eruption. In this particular it differs from the petechiæ which occur in the advanced stage of many fevers, and cannot be considered essential to them. "The petechiæ," says Bruserius,§ "besides that they break out in all patients, or at any rate in by far the greatest number, as I have already said, likewise appear sooner in particular instances, generally about the fourth day, sometimes even earlier; but very seldom if ever at all delay breaking out beyond the seventh day, unless they be very anomalous, while the secondary and symptomatic ones appear much seldomer, and in fewer patients, nay, very late," &c.

---

of the poison carried through the circulation to the nervous centres, or whether, as Dr. Hodgkin infers, from a conversion of Edwards' proposition, "since cold has the effect of retarding, especially that function by which particles to be rejected from the body are thrown off, *a suspension of this process from another cause should be attended with a sensation resembling in a degree those caused by cold*." This seems rather a doubtful conversion of Dr. Edwards' fact. I shall hereafter return to Dr. Hodgkin's ingenious speculations, merely observing for the present, that while some parts (see page 491) support a humoral theory, his theory will by no means explain the phenomena of *infection* as the humoral theory does.

* Ratio Medendi. Vol. II. Chap. I.

† Edinburgh Medical and Surgical Journal. Vol. XXVIII.

‡ Idem. Vol. XLVII. § Institutes. Vol. III.

Hoffman* also describes them as appearing "in nonnullis *quarto* vel *circa septimum* diem in dorso potissimum pectore et brachiiscum vel sine levamine maculæ in aliis copiosiores in aliis pauciores coloris varii," &c. Modern observations are consistent with these. Thus, Dr. Barker, after taking much pains to prove by a reference to older authors, that this eruption was not peculiar to the Irish epidemic of 1817-18, says, "From a comparison of many cases, I would infer that it generally makes its appearance between the fifth and seventh days inclusive of the fever," &c.†

If we refer to descriptions of the jail or hospital fever, we find Monro enumerating the fourth, fifth, sixth, and seventh as the most frequent days of the measly eruption; and Sir J. Pringle states that he frequently saw them as early as the fourth or fifth day.‡

Another resemblance is presented in the phenomena attending the progress of the disease; more especially the attenuation which may be observed between the eruption and the affections of mucous membranes. In exanthematous typhus the same dry harassing cough is observed previous to the appearance of the eruption as in measles. On the coming out of the eruption this subsides, unless a catarrhal complication exists. Again, if the mucous membrane of the bowels be the seat of irritation,and a diarrhœa (whether the effect of the disease or of medicine exist) the eruption will fade. This is analogous to what has been observed in scarlatina,§ and it has been urged as an argument for the free use of purgatives in typhus, that they clear the skin from spots. In the definite nature of its progress, and its disposition to terminate critically and at once, typhus resembles the exanthemata as much as it differs from the intermittent and remittent fevers with which it has been confused and compared. Neither does it appear that when once the febrile movement has commenced it can be arrested any more than the action of other morbid poisons. Most of the cases in which this is supposed to have been done have been merely cases of strong nervous shock from exposure to infection, without evidence of the infection having been imbibed into the system.

The last resemblance upon which it is necessary to dwell, is the mode of communication.

The fact of typhus being communicated from one person to another, is a powerful argument for classing it among the *special contagions*. An examination (hereafter) of the circumstances which *favour* infection, will shew them to be the same in both, and the time at which they become infectious seems to be the same in both, viz. at and after the period of maturation or crisis. The argument adduced by Dr. Ferriar against the humoral theory, "that neither would a patient after recovering from a nervous fever, cease to infect others till the whole mass of his fluids were changed," is thus deprived of its weight.

The histories of patients admitted into our fever hospital afford frequent illustrations of this fact, as they constantly attribute their infection to some neighbour, or member of their family, who has returned home cured from hospital; and there is at present in the hospital a man who has suffered severely from this cause, having lately lost his wife by a typhous fever which commenced on one of his children, who was hugged and kissed by a man upon his discharge from the hospital after passing through a most severe typhus.

But as evidence on a large scale is to be preferred to individual instances, let us take Dr. Perry's very strong and satisfactory testimony to the fact with reference to *both* diseases. ||

Into the fever house in Glasgow are admitted cases of measles, scarlatina and small-pox, and patients are very frequently sent in labouring under bronchitis,

---

* Medicinæ Rationalis. Tom IV. p. 120.

† Dublin Medical Transactions. Vol. II.

‡ Monro on Hospitals, page 10; and Sir J. Pringle on Diseases of the Army, page 299.

§ By Fothergill, and others. || Dublin Medical Journal. Vol. X.

&c. &c. I found by experience that when the latter class of patients were sent into the convalescent ward, where they necessarily mixed with the others, almost all who had not previously had typhus fever were either seized with it before leaving the house, or returned soon after labouring under it. The period intervening between the time of their being sent to the convalescent ward and the attack being never less than eight days. Although means were taken to keep those recovering from small-pox, scarlatina, &c. in a separate room from those convalescing from fever, the rooms being adjoining the non-intercourse was incomplete, and the result was, that these diseases occasionally spread among the typhous convalescents, and the convalescents from small-pox and scarlatina caught typhus." He states that "the result of a trial of the plan of keeping non-febrile cases in the acute wards till able to go to their homes was, that *not one* so detained ever caught fever in the wards, or returned with it afterwards." Dr. Perry's statement is confirmed by Dr. Stewart, who says, "In fact, scarcely one of the hundreds dismissed from the *acute* wards ever returned labouring under typhus, though they had remained for a week or ten days in wards sometimes crowded to excess, while of the few who by mistake went into the *convalescent* wards, scarcely one escaped the disease, and several died." *

Such are some of the most striking analogies between typhus and the class exanthemata; others not less important arise from a consideration of the supposed discrepancies which exist between the laws and phenomena of the two diseases.

Each writer who has opposed this classification of fever, has urged some objection or other which he considered fatal to it. We shall examine them in detail, and endeavour to show that they belong to two classes. 1. Those which apply to the exanthemata as well as to typhus; and 2. Those which *do not* apply to typhus, but to other fevers.

In both cases the argument from discrepancy must be ill-founded, as in the first the differences become anologies, and in the second, typhus, by being separated from other fevers, becomes more completely identified with the "specific contagions."

To commence with the latent period of typhus. Its variable length has been urged against the classification. That of the exanthemata appears to be equally so. In scarlatina it may extend from a few hours to twenty-one days, according to Dr. Williams and Dr. Maton. In measles from a week to a fortnight, and in small-pox from five to twenty-three days.

II. The eruption, it is said, is not invariably present. This objection is not as strong as it appears, and since it is admitted that the eruption of typhus has only very lately been attentively examined as a diagnostic character of the disease, we cannot think the question likely to be illustrated by the kind of testimony which some opponents bring to bear upon it. †

The answers to this objection are, 1. It is often present, though so indistinct as to escape a superficial examination. "On such occasions," says Dr. Barker, ‡ "*the suffusion of the eyes* is a pretty certain indication of its presence." "They sometimes," says Bruserius, § "lurk under the epidemics, scarcely perceptible, and are only seen through it on attentive examination; nay, they sometimes do not appear unless cupping glasses be applied, by which they are called out."

Similar is the observation made by Sir J. Pringle, || and repeated by Dr. Roupell, upon the arm on which a ligature had been applied for bleeding.

---

* Edinburgh Med. and Surg. Journal, No. CXLV.

† *Vide* Dr. West's paper.

‡ Dublin Medical Transactions, Vol. II. Monro also remarks, that though many had no petechiæ, in all who were very bad the countenance looked *bloated*, and the *eyes reddish and somewhat inflamed*, page 12.

§ Institutes. || Page 300.

2. In the returns from which the comparative frequency of appearance of the eruption is deduced, there are two sources of error which have been well exposed by Dr. Davidson. The first is, that they contain a large proportion of cases *not* typhus; the other, that many of them entered hospital at an advanced stage of the disease, after the retrocession of the eruption.

Dr. Davidson observes that one fact powerfully supports the opinion that contagious typhus, in the great majority of cases, particularly in adults, is attended with the eruption, viz. that almost all the instances of fever which have occurred during the last six or seven years among the physicians, clerks, nurses, &c. of the Glasgow fever-hospital, have been accompanied with this exanthema. *

The following remarks of Dr. Stewart on this subject deserve consideration.

"Nor can I consent without reserve to conclusions drawn from the alleged absence of eruption; for the fact I have already referred to (viz. that the eruption in typhus in Edinburgh was unheeded before 1832) shews how appearances may escape the eye of the most distinguished and practised physicians, when their attention is not particularly drawn to them. It is also well-known to many, that previous to a visit which Dr. Peebles made to the Glasgow fever-hospital, in the spring of 1835, the exanthema of typhus, then found to be of general occurrence, had neither been looked for nor registered in that institution, and was received as a new discovery." †

3. We reply that the occasional absence of the eruption is in truth an *analogy*. "For," says Burserius, "as the variolous fever, or the variolous disease unaccompanied with small-pox, sometimes occurs, I should not consider it at all absurd to suppose that the petechial fever may in like manner take place without petechiæ."

In another place this author remarks: "This is generally observed to happen when they prevail epidemically. But it does not occur so frequently and decidedly to the observation of any one as that of the inoculators. For not unfrequently at the usual time after the inoculation, a fever comes on which continues several days, and then goes off without being followed by an eruption of pustules. Who would not call it a variolous fever? ‡

I am acquainted with a family in which small-pox made its appearance, affecting different individuals in the following modes. One with confluent eruptions, another with scanty, two with variolous fever without eruption, and another with intense vomiting and delirium, but no subsequent fever or eruption.

The same occurrence of a peculiar fever without eruption, has been remarked in epidemics of measles, by Sydenham and others. Rayer states that Guersent has observed individuals in families where measles prevailed, exhibiting all the other symptoms of the disease except the eruption, and that he has himself several times seen cases in which the eruption was incomplete, and which might have been referred to the morbillary fever of Sydenham. §

Every one who has had any experience of epidemics of scarlatina, must have observed fever and sore throats of the same character as that of scarlatina, but without eruption, occurring in families in which this disease prevailed. Rayer quotes the testimony of a number of authors upon the subject, and Dr. Tweedie introduces it as a variety of the disease into his classification. This scarlatina sine exanthemate is very frequently met with in practice.

III. A want of uniformity of the character and time of appearance of the eruption has been alleged.

---

* Essay. page 22. † Edinburgh Med. and Surg. Journal, Vol. LIV.
‡ Institutes, Vol. III.
§ On Diseases of the Skin.

"Of the varying characters of the eruption," says Dr. West, * "almost every quotation has afforded an illustration, and we have seen the date of its appearance vary from the second to the seventeenth day."

We are by no means convinced that the subject has been *illustrated* by Dr. West's quotations, which appear to be descriptive not so much of typhus as of every other variety of fever. On the other hand, testimony is not wanting of observers who have explained these apparent irregularities in the character and periods of the typhus eruption, and reconciled their apparent inconsistency with an exanthematous theory of fever.

Such we meet in the following passage from Burserius's admirable chapter on petechial fever. "Le Roy also observes that there is some distinction between the primary and secondary petechiæ, which consists in the difference of their colour, namely, that the former are of a palish red and rosy colour, and in general break out in great numbers, principally on the loins and legs, that the latter on the contrary are generally of a purple colour, like deep red wine, and are sometimes also brown or black, and fewer in number."

But we must also remember that the primary ones break out soon, and when they are epidemic appear not only in all affected with the same disease, but are likewise very frequently combined with other diseases called intercurrent ones —(for these last are not always wanting, as some contend)—while on the other hand, the secondary ones break out later, and generally about the height or towards the end of the disease, and not in all patients, but only in those whose blood is so vitiated as to become almost putrid and occasion gangrenes here and there on the skin, or being thrown into violent commotion by a heating regimen and medicines, is effused into the spaces of the skin, *but not by the wisdom of Nature endeavouring to free herself from the noxious miasma.* Hence I would say that the primary differ from the secondary petechiæ, because the former arise from a peculiar and poisonous miasma, and the secondary from the crasis of the blood being deranged by the violence of the disease, or from its increased motion, or lastly, from a heating regimen having been employed." Such also we meet in Dr. Staberoh's paper on the eruption attending epidemic fever. In which he shows that not only do petechiæ of the ecchymotic or secondary kind occur after and *apart* from the exanthema, but that spots of these are capable of being converted into ecchymotic spots.† Attentive observation has convinced me that not only are the above statements correct, but that we may add that a third variety of late petechiæ occur in cases in which from diarrhœa or hypercatharsis in the beginning of fever the exanthema lurked under the epidemics. The conversion of this *indistinct eruption* into ecchymosis taking place, or the latter being superadded in the course of the disease, *and appearing to be primary.* A fourth variety is thus alluded to by Dr. Peebles; "Petechiæ may be mixed with the exanthema, and in some epidemics the exanthema has been prevented from showing itself by the disease passing so rapidly from the sthenic state to the putrid, that it has not had time to make its appearance."

Of course under any of the foregoing circumstances the late appearance of a petechial eruption is no argument for a want of uniformity in that of the exanthema. The frequency of occurrence of these secondary petechiæ is only an additional reason for believing that the two forms have been by many writers confounded together.

IV. It is objected, "That the disease often occurred more than once during the life-time of an individual."

---

* On Exanthematous Fevers. Edinburgh Med. and Surg. Jour. No. CXLIII.
† London Medical Gazette, Vol. I. N. S. p. 973.

