# Errata

**Published:** 1841-10-01

**Authors:** 


### ERRATA.

Page 1—line 8, *for* account, *read* amount.
,, 9—line 30, *after* on the, *insert* contrary.
,, 14—line 32, *for* violent, *read* violet.
,, 17—line 17, *for* committed, *read* admitted.
,, 24—line 9, *for* receiving, *read* secerning.
,, 24—line 23, *for* phelibilis, *read* phlebitis.
,, 24—line 48, *for* universally, *read* inversely.
,, 28—line 13, *for* Brusenis, *read* Burserius.
,, 29—line 14, *for* alteration, *read* alternation.
,, 30—line 45, *for* epidemics, *read* epidermis.
,, 32—line 36, *for* epidemics, *read* epidermis.

